# Supplementary material for: Does Body Mass Index Impact the Clinical Response to Dupilumab Therapy in Atopic Dermatitis? A Monocentric Study of 170 Patients
Source: J Clin Med. 2024 Aug 5;13(15):4559. doi: 10.3390/jcm13154559 (PMC11313165; doi:10.3390/jcm13154559)
Supplement: Supplementary file 1 [file jcm-13-04559-s001.zip › jcm-3084981-supplementary.pdf]

Table S1

Score	BMI Group	T0	T4	T0-T4	T16	T0-T16	T32	T0-T32	T48	T0-T48
EASI (n =170)	BMI < 25	26.0 (24.0-30.0)	6.0 (3.0-10.0)	< 0.001	3.0 (1.0-6.0)	< 0.001	2.0 (1.0-4.0)	< 0.001	2.0 (1.0-4.0)	< 0.001
	BMI ≥ 25	26.0 (24.0-30.8)	7.0 (2.0-12.0)	< 0.001	4.0 (2.0-7.8)	< 0.001	3.0 (1.0-5.0)	< 0.001	2.0 (1.0-5.0)	< 0.001
Pruritus NRS (n=170)	BMI < 25	9.0 (8.0-10.0)	3.5 (2.0-5.0)	< 0.001	3.0 (1.0-5.0)	< 0.001	3.0 (1.0-4.0)	< 0.001	3.0 (1.0-4.0)	< 0.001
	BMI ≥ 25	9.0 (8.0-10.0)	4.0 (2.0-6.0)	< 0.001	2.0 (1.0-5.0)	< 0.001	2.0 (1.0-5.0)	< 0.001	2.0 (1.0-5.0)	< 0.001
Sleep NRS (n=170)	BMI < 25	8.0 (6.0-10.0)	1.0 (0.0-4.0)	< 0.001	0.0 (0.0-3.0)	< 0.001	0.0 (0.0-1.5)	< 0.001	0.0 (0.0-0.0)	< 0.001
	BMI ≥ 25	7.0 (4.3-9.0)	1.0 (0.0-3.0)	< 0.001	0.0 (0.0-1.8)	< 0.001	0.0 (0.0-1.0)	< 0.001	0.0 (0.0.-0.75)	< 0.001
POEM (n=170)	BMI <2 5	23.0 (18.0-26.0)	8.0 (4.5-12.0)	< 0.001	7.5 (3.0-13.0)	< 0.001	5.0 (3.0-8.8)	< 0.001	6.0 (3.0-9.3)	< 0.001
	BMI ≥ 25	23.5 (20.0-28.0)	7.5 (5.0-13.3)	< 0.001	5.0 (3.0-10.8)	< 0.001	5.0 (2.0-11.3)	< 0.001	5.0 (2.0-10.0)	< 0.001
ADCT (n=160)	BMI < 25	20.0 (16.8-23.0)	6.0 (4.3-9.0)	< 0.001	4.0 (3.0-7.0)	< 0.001	4.0 (2.0-6.0)	< 0.001	3.0 (1.0-5.0)	< 0.001
	BMI ≥ 25	21.5 (18.0-24.0)	7.0 (6.0-10.0)	< 0.001	6.0 (4.0-8.0)	< 0.001	4.0 (2.0-7.0)	< 0.001	4.0 (2.0-7.0)	< 0.001
DLQI (n=170)	BMI < 25	15.0 (11.0-20.25)	3.0 (1.8-8.3)	< 0.001	3.0 (1.0-7.0)	< 0.001	2.0 (1.0-5.0)	< 0.001	2.5 (1.0-5.3)	< 0.001
	BMI ≥ 25	14.0 (10.0-20.0)	6.0 (3.0-9.0)	< 0.001	2.0 (1.0-5.0)	< 0.001	3.5 (1.0-6.8)	< 0.001	1.5 (1.0-3.8)	< 0.001

Table S1. An assessment of EASI, pruritus NRS, sleep NRS, POEM, ADCT, and DLQI, stratified by BMI group, at different time points. The absolute scores are represented as the median (Q1-Q3). The improvement at different time points was statistically tested using the Wilcoxon test. Abbreviations: EASI, Eczema Assessment Severity Index, BMI, body mass index; POEM, Patient-Oriented Eczema Measure; DLQI, Dermatology Life Quality Index; ADCT, Atopic Dermatitis Control Tool; NRS, Numerical Rates Scale; T, time point in weeks.

Table S2

Score	Follow-up	BMI < 25	BMI ≥ 25	p-value
EASI (n =170)	T0 (absolute score)	26.0 (24.0-30.0)	26.0 (24.0-30.8)	p=0.909
	Improvement (%) T0-T4	79.2 (62.0-88.1)	75.5 (65.2-85.4)	p=0.660
	Improvement (%) T0-T16	88.2 (76.7-96.0)	86.6 (77.0-93.2)	p=0.264
	Improvement (%) T0-T32	92.0 (87.5-96.7)	89.3 (82.4-95.8)	p=0.058
	Improvement (%) T0-T48	94.3 (87.5-97.5)	92.4 (81.4-96.9)	p=0.363
Pruritus NRS (n =170)	T0 (absolute score)	9.0 (8.0-10.0)	9.0 (8.0-10.0)	p=0.637
	Improvement (%) T0-T4	60.0 (39.4-77.8)	53.6 (30.0-77.8)	p=0.261
	Improvement (%) T0-T16	70.0 (37.5-87.5)	70.7 (55.6-87.5)	p=0.587
	Improvement (%) T0-T32	70.0 (52.8-88.9)	75.0 (41.1-90.0)	p=0.888
	Improvement (%) T0-T48	70.0 (50.0-90.0)	75.0 (34.4-90.0)	p=0.790
Sleep NRS (n =170)	T0 (absolute score)	8.0 (6.0-10.0)	7.0 (4.3-9.0)	p=0.06
	Improvement (%) T0-T4	88.9 (53.6-100.00)	85.7 (50.0-100.0)	p=0.625
	Improvement (%) T0-T16	100.0 (64.6-100.0)	100.0 (71.4-100.0)	p=0.477
	Improvement (%) T0-T32	100.0 (83.9-100.0)	100.0 (77.8-100.0)	p=0.641
	Improvement (%) T0-T48	100.0 (100.0-100.0)	100.0 (71.-100.0)	p=0.472
POEM (n =170)	T0 (absolute score)	23.0 (18.0-26.0)	23.5 (20.0-28.0)	p=0.233
	Improvement (%) T0-T4	61.1 (30.8-79.6)	60.7 (41.3-81.9)	p=0.943
	**Improvement (%) T0-T16**	**63.6 (38.8-81.6)**	**78.8 (54.7-86.6)**	**p=0.013**
	Improvement (%) T0-T32	75.0 (55.6-86.4)	71.4 (44.2-90.7)	p=0.969
	Improvement (%) T0-T48	72.1 (50.0-84.9)	76.3 (58.2-89.3)	p=0.170
ADCT (n =160)	**T0 (absolute score)**	**20.0 (16.8-23.0)**	**21.5 (18.0-24.0)**	**p=0.043**
	Improvement (%) T0-T4	64.9 (50.7-75.0)	62.5 (47.8-72.7)	p=0.410
	Improvement (%) T0-T16	73.9 (60.0-83.3)	71.4 (55.6-80.0)	p=0.225
	Improvement (%) T0-T32	81.3 (70.0-92.3)	77.3 (63.6-91.7)	p=0.445
	Improvement (%) T0-T48	81.8 (71.4-92.3)	76.5 (63.2-91.3)	p=0.134
DLQI (n =170)	T0 (absolute score)	15.0 (11.0-20.25)	14.0 (10.0-20.0)	p=0.507
	**Improvement (%) T0-T4**	**73.7 (42.7-90.2)**	**59.0 (28.0-79.9)**	**p=0.022**

Score	Follow-up	BMI < 25	BMI ≥ 25	p-value
	**Improvement (%) T0-T16**	**73.6 (45.3-88.9)**	**89.2 (71.2-95.0)**	**p=0.002**
	Improvement (%) T0-T32	83.3 (61.7-92.8)	80.0 (50.0-93.1)	p=0.462
	Improvement (%) T0-T48	83.3 (62.4-93.3)	87.5 (74.0-96.1)	p=0.100

Table S2. Assessment of the percentage improvement of EASI, pruritus NRS, sleep NRS, POEM, ADCT and DLQI, compared between the two BMI groups, at different time-point. The percentage improvement is represented as median (Q1-Q3). The differences among the two BMI group were tested using the Mann-Whitney U test. Abbreviations: EASI, Eczema Assessment Severity Score; BMI, body mass index; POEM, Patient-Oriented Eczema Measure; DLQI, Dermatology Life Quality Index; ADCT, Atopic Dermatitis Control Tool; NRS, Numerical Rates Scale; T, time-point in weeks.

Table S3

Covariates	Single regression analysis				Multivariate regression analysis			
	p-value	OR	95% C.I. (inferior)	95% C.I. (superior)	p-value	OR	95% C.I (inferior)	95% C.I. (superior)
**BMI (BMI ≥ 25 vs. BMI < 25)**	0.109	2.467	0.818	7.436	0.138	2.613	0.734	9.299
**Atopic comorbidities**	0.439				0.505			
1 atopic comorbidity vs. 0	0.216	0.241	0.025	2.297	0.243	0.240	0.022	2.629
2 or more atopic comorbidities vs. 0	0.426	0.601	0.172	2.104	0.612	0.694	0.169	2.846
**Atopic family history (positive vs. negative)**	0.158	0.421	0.127	1.400	0.145	0.382	0.105	1.394
**AD onset age (adult-onset vs. childhood-onset)**	0.602	1.383	0.409	4.679	0.414	0.516	0.105	2.525
**Sex (female vs. male)**	0.171	0.432	0.130	1.436	0.304	0.506	0.138	1.853
**Systemic drug at baseline (not intake vs. intake)**	0.173	2.175	0.711	6.652	0.093	0.330	0.090	1.202
**Phenotype**	0.172				0.420			
Phenotype other than PN or classic vs. classic	0.322	1.902	0.534	6.782	0.251	2.182	0.576	8.267
PN vs. classic	0.061	4.687	0.931	23.597	0.301	2.653	0.417	16.861
Constant					0.188	0.223		

Table S3. Single and a multivariate regression analysis evaluating a potential predominating factor for the determination an EASI improvement inferior to 75% (EASI-75) at T48. The following covariates were considered: BMI group (BMI≥25 vs. BMI<25), sex (female vs. male), onset age (adult-onset vs. child-onset or adolescent-onset), atopic comorbidities (2 or more comorbidities vs. 0 comorbidities and 1 comorbidity vs. 0 comorbidities), AD phenotype (prurigo nodularis [PN] vs. classical phenotype and phenotypes other than classical and PN vs. classical), intake of systemic drug at baseline among cyclosporin, methotrexate or systemic corticosteroids (no drug intake vs. drug intake) and atopic family history (positive vs. negative). Abbreviations: BMI, body mass index; T0, baseline; AD, atopic dermatitis; OR, odd ratio; C.I.., confidence interval.
